# Spelling Proficiency of Children with a Resolved Phonological Speech Sound Disorder Treated with an Integrated Approach—A Long-Term Follow-Up Randomized Controlled Trial

**DOI:** 10.3390/children10071154

**Published:** 2023-06-30

**Authors:** Denise I. Siemons-Lühring, Amélie E. Hesping, Harald A. Euler, Lars Meyer, Corinna Gietmann, Boris Suchan, Katrin Neumann

**Affiliations:** 1 Department of Phoniatrics and Pedaudiology, University Hospital Münster, University of Münster, Malmedyweg 13, 48149 Münster, Germany; amelieelisabeth.hesping@ukmuenster.de (A.E.H.); euler@uni-kassel.de (H.A.E.); katrin.neumann@uni-muenster.de (K.N.); 2 Max Planck Institute for Human Cognitive and Brain Sciences, Stephanstraße 1A, 04103 Leipzig, Germany; 3 Department of Clinical Neuropsychology, Ruhr-University of Bochum, Universitätsstraße 150, 44801 Bochum, Germany

**Keywords:** speech sound disorder, phonological, treatment, therapy, spelling disorder, dyslexia, reading disorder, RCT, follow-up

## Abstract

Phonological developmental speech sound disorders (pDSSD) in childhood are often associated with later difficulties in literacy acquisition. The present study is a follow-up of the randomized controlled trial (RCT) on the effectiveness of PhonoSens, a treatment for pDSSD that focuses on improving auditory self-monitoring skills and categorial perception of phoneme contrasts, which could have a positive impact on later spelling development. Our study examines the spelling abilities of 26 German-speaking children (15 girls, 11 boys; mean age 10.1 years, range 9.3–11.2 years) 3–6 years after their successful completion of the PhonoSens treatment. Spelling assessment revealed that only 3 out of 26 participants developed a spelling disorder. In the overall population of fourth-graders, one in five children showed a spelling deficit; in another study of elementary school children, with resolved pDSSD, 18 of 32 children had a spelling deficit. Thus, the applied pDSSD treatment method appears to be associated with positive spelling development. Multiple regression analysis revealed that among the potentially predictive factors for German-speaking children with resolved pDSSD to develop later spelling difficulties, parental educational level and family risk for developmental language disorder (DLD) had an impact on children’s spelling abilities; gender and the child’s phonological memory had not.

## 1. Introduction

Phonological developmental speech sound disorders (pDSSD) (ICD-11 6A01.0, World Health Organization 2021) are among the most common communication disorders in preschool children. They are often associated with subsequent deficits in reading (ability to convert letters or visual signs into meaningful words) and spelling development (ability to correctly convert phonemes into graphemes) at school age [1,2,3], which can compromise educational success and social status [4]. Although most pDSSD are treated successfully before school entry (so-called ‘resolved pDSSD’), underlying cognitive-linguistic deficits resulting from inadequate phonological language processing can impede later reading and spelling acquisition (here defined as literacy acquisition) [5,6,7]. Co-occurring risk factors for literacy deficits include poor lexical-semantic or morphological-syntactic skills in a child or family predisposition [8,9,10]. Language-specific characteristics may also influence later reading and spelling development, which became apparent in a study by Landerl and colleagues [11]. In their study, the same underlying phonological processing deficits were found, e.g., in the Spoonerism task (substitution of word-initial phonemes or morphemes), in English and German children with literacy deficits. Yet, the English children showed more deficits in reading acquisition, while the German children had more deficits in spelling acquisition. In line with this, a German education report showed that out of 29,259 fourth-graders, 22.1% failed to meet the minimum standards <1 SD (standard deviation below the mean) in spelling proficiency, but only 12.5% failed to meet the minimum standards in reading proficiency [12,13]. The nature of reading deficits also differs between languages. German children with reading deficits were found to have difficulties, especially in fluent reading, whereas English children with reading disorders mainly had difficulties in reading unknown words correctly [14].

One reason for the language differences in literacy acquisition could be differences in phoneme-to-grapheme correspondence (i.e., transparent versus shallow orthography). Forward regularity describes how consistently the same letters are pronounced the same way in different words. Backward regularity describes how consistently phonemes can be assigned to graphemes in different words. If a language has high regularity, phonological skills will have less influence on literacy acquisition; in contrast, phonological deficits will cause problems in the literacy acquisition of a language with low consistencies [11]. The following example shows how challenging phoneme-to-grapheme mapping can be. In order to read and pronounce the English words <nation> or <national> correctly, the words must first be understood completely in order to deduce how the vowel <a> in the syllable <na> is pronounced: [′neɪ∫n] or [′næ∫nəl]. In German, high forward regularity (84% for monosyllabic words) facilitates reading acquisition. The low backward regularity of German (47%) may, however, lead to significant problems in spelling development [14]. Since spelling development is more likely to cause problems for German-speaking children than reading development, we focus on spelling acquisition in the study presented here.

In the literature, various hypotheses are put forward as causes of deficits in written language development. These include the phonological deficit hypothesis, which causally assumes deficits in the processing of phonemic language units [15,16]. Impaired phonological awareness and impaired verbal short-term memory are also constituents of the phonological deficit hypothesis [17]. In the double deficit hypothesis, deficits in rapid naming are assumed contributions to phonological processing deficits as an additional factor (e.g., [18]). 

Another theory is that of orthographic deficit, which is the impaired ability to recognize letter patterns and words as whole units. This ability can be more broadly related to a presumed selective impairment in procedural learning and control of established sensorimotor and cognitive habits, skills, and procedures [19]. These include implicit sequence learning [20,21] and probabilistic category learning; skills that also play an important role in speech sound development [22]. However, the exact relationship between impaired procedural learning and phonological deficits is still unclear [23].

The number of follow-up studies investigating the subsequent spelling ability of children with resolved isolated pDSSD (here, isolated means that a developmental language disorder [DLD] was restricted to a pDSSD) is small, and comparability across studies is restricted since the influence of previous pDSSD treatments on children’s later acquisition of written language is often difficult to measure retrospectively, e.g., because of imprecise or absent descriptions of the treatment methods or doses applied, heterogeneous treatment strategies, and other confounding factors.

### 1.1. Spelling Acquisition

Spelling development follows the structure of the orthographic processes of a given language, integrating sub-lexical processes (direct conversion of spoken phonemes into their associated graphemes) and lexical processes (assessment of word-specific orthographic representations) [24]. In the Hamburg Writing Test (German: Hamburger Schreib-Probe = HSP), a common test to assess the German spelling competence of first- to tenth-graders, these processes are referred to as spelling strategies [25]. The strategies include sub-strategies (here called alphabetical, orthographic, morphemic, and across-word strategies) that are rule-guided and systematically linked to each other. A child’s individual orthographic skills can be determined not only by the number of correctly written words and graphemes but also by the analysis of the spelling strategies used.

At the beginning of spelling acquisition, the alphabetic spelling strategy (sub-lexical process) is developed, in which phonetic spelling is controlled by the spoken word, and the individual phonemes are assigned to graphemes. However, the German language has a high phoneme-grapheme inconsistency (53% for monosyllabic words) [14]. This means that, in many cases, a phoneme can be represented by different graphemes or even grapheme combinations. For example, the phoneme /∫/ is usually written as <sch> in German, but as <st> and <sp> in the consonant clusters /∫t/ and /∫p/. Similarly, the diphthong /aɪ/ can be represented graphemically by <ei> in <Seite> (page) or as <ai> in <Saite> (cord). Orthographic spelling strategies require the application of specific spelling rules or the implementation of a specific phoneme-grapheme relationship for a particular sound. This includes, for example, consonant doubling after a short vowel in German orthography, as in <Kuss> (kiss). Knowledge of inflectional and derivational endings must be used in the morphemic spelling strategy, as is evident in the spelling of a final <d> in the word <Hund (dog), even though the word is pronounced [hunt]. Since the plural is pronounced as [hundə], the written word <Hund> requires a final <d> in the singular form. The across-word strategy derives orthographic rules from the context of the word and sentence. For example, verbs are normally written in lowercase, but in the function of a noun, the verb is capitalized [25]. 

### 1.2. pDSSD and Later Spelling Skills

The literature is equivocal on whether isolated pDSSD is a risk factor for reading or spelling deficits [5,8,26,27]. However, electrophysiological studies in particular strongly suggest such a relationship and there is robust evidence for difficulties in phoneme discrimination and later impaired literacy [28,29,30,31]. For English-speaking children, only weak evidence has been found for an association between isolated pDSSD and later spelling deficits [9,32]. In contrast, for German-speaking children, there is evidence that both resolved isolated pDSSD and resolved pDSSD in combination with other (lexical-semantic or morphological-syntactic) language deficits, have a negative impact on spelling development [10,26].

Schnitzler [27] examined the reading and spelling abilities of 48 primary school-aged, German-speaking children with resolved pDSSD, who had completed an unspecified speech therapy prior to school entry. Two control groups, one for reading and one for spelling skills, of 48 children each, were matched for gender, age, and school age. Fifty-six percent of children with resolved isolated pDSSD (n = 32) had a spelling deficit, and for children with resolved pDSSD including other language deficits (n = 16), the percentage was as high as 75%; among controls with no history of speech therapy, only 21% did.

The study presented here focuses on a group of children with resolved isolated pDSSD. In the Schnitzler study, a group with resolved isolated pDSSD was further subdivided based on phonological processes evident in the earlier pDSSD into a group with phonological delays (n = 10) and one with pathological phonological processes (n = 22). Phonological processes are patterns of speech sound errors that children temporarily use to simplify speech during typical language development. Phonological disorders occur when phonological processes persist beyond the period at which most typically-developing children stop using them, usually six months longer persistence (physiological processes), or when phonological processes that do not occur during typical language development are used regularly (pathological processes) [33]. Phonological disorders can therefore be divided into phonological delays characterized by persistent physiological phonological processes with consistent word production, and phonological disorders with pathological (or non-physiological) phonological processes and either consistent or inconsistent word production [34]. Due to the small number of participants in each subgroup, we consider the children with resolved isolated pDSSD from the Schnitzler study collectively (n = 32) and use this group for a descriptive comparison to the children with resolved isolated pDSSD examined here.

The extent to which children with resolved pDSSD succeed in developing appropriate spelling strategies can be influenced by several factors, including the type of therapy they have received [35]. Interventions that focus solely on the revision of phonological processes in spoken language appear to be insufficient for successful written language acquisition [36,37], whereas therapy that focuses on the perception of individual phonemes seems to be advantageous [26,27,38]. If written language elements such as letters or speech sound symbol pictures (SSP cards) are used in therapy to identify individual phonemes, this could also have a positive effect on later spelling acquisition [35].

### 1.3. Categorical Phoneme Perception and Literacy Acquisition

In order to better understand the importance of the phonemic level, the role of categorical phoneme perception is explained in more detail below. Categorical perception describes the phenomenon in which certain stimuli along a continuum (e.g., speech sounds) are perceived categorically rather than continuously. Furthermore, categorical perception distorts the perceptual space, so that elements are perceived as more or less similar than their actual acoustic distance would suggest. In this way, phoneme categories emerge that have a word-discriminating effect. Children with pDSSD often demonstrate difficulties in categorical speech perception [39,40].

Speech sounds that represent a category are called phonemes, and individual expressions of a single phoneme are called allophones [41]. How speech sounds are grouped into allophones or phonemes depends on the particular language being examined and can be explored through the formation of language-typical minimal pairs. While in English the minimal pairs [maʊθ] and [maʊs] differ in meaning (mouth and mouse), the same words in German would be two variants of one word (mouse lisped and mouse pronounced correctly) with no difference in meaning. Thus, in English, [θ] and [s] are two different phonemes, but in German, they are two allophones [41].

During language development, discrete, meaningful phoneme categories (and thus phonemic contrasts) are acquired through exposure, i.e., a child learns to distinguish the phonemes of the surrounding language and to identify the associated allophones. For later acquisition of written language, phonemic contrasts must be adequately mapped to graphemes [42,43]. Children with literacy deficits tend to do better at discriminating within a category (allophonic), whereas children without literacy deficits do better at discriminating between categories (phonemic) [37]. This may lead to inadequate phoneme-grapheme mapping. For example, Pennala and colleagues found a clear relationship between the quality of the categorical perception of phoneme length and the spelling skills of second-graders [44]. Therapy that focuses on the perception of individual phonemes therefore might be advantageous over treatment approaches that target only the revision of phonological processes.

### 1.4. Additional Risk Factors for Spelling Deficits

Deficits in written language acquisition and DLD are common comorbidities, both are thought to have a genetic component, with phonological deficits being a unifying feature [45]. Therefore, a family history of DLD could be a risk factor for literacy acquisition [5,6,7]. Deficits in short-term phonological memory for non-word repetitions have also been identified as a predictor of reading and spelling disorders [46]. The ability to represent and store new and previously unheard speech sound patterns in phonological memory for a short period of time is age-dependent, increases steadily between three and eight years of age [47], and can be tested from three years of age. A lack of phonological awareness is also considered a risk factor for literacy acquisition. However, phonological awareness is also trained by reading acquisition [7] and therefore cannot be measured retrospectively as a predictor for spelling acquisition. Only a baseline measure of phonological awareness prior to the onset of reading acquisition could have been used as a predictor here. However, for the children studied here, no testing of phonological awareness took place prior to their initial start of speech therapy, as only norm-referenced testing of phonological working memory was available for children in the age range of 3.5 to 5.5 years. Other risk factors identified in the study assessing the reading and spelling skills of German fourth-graders were the influence of parental educational level [48] and gender (boys are at higher risk for a spelling disability) on spelling acquisition [12].

### 1.5. Aims of the Study

This study is a follow-up study to an RCT on the effectiveness of the PhonoSens integrated treatment method in German-speaking children with pDSSD [49]. The aim was to examine the later spelling abilities of the former RCT participants (all of whom had successfully overcome their pDSSD during the RCT) three to six years after enrollment. Furthermore, the potential influence of parameters that have been shown to be predictive of later spelling skills, i.e., parental educational level, family predisposition to DLD [50], gender [12], and children’s pretreatment phonological working memory performance for non-word repetition [47], is examined.

As a secondary outcome, elementary school recommendations for secondary school attendance were used as indicators of later educational success.

### 1.6. Hypotheses

The children investigated in this study received treatment with PhonoSens and overcame their pDSSD either before school enrollment or during first grade. PhonoSens is among the therapies that focus on the perception of individual phonemes, use speech sound symbol pictograms (SSP cards) to identify individual phonemes (such as a dripping tap for [t]), and strengthen auditory self-monitoring skills, which might have a positive impact on later spelling acquisition [32]. However, there are other risk factors that may influence spelling success. We, therefore, hypothesized the following:

**H1-1:**  Three to six years after school enrollment, children with resolved pDSSD who have received speech therapy with PhonoSens demonstrate better spelling skills than the children examined in Schnitzler’s study [27].

**H1-2:**  Three to six years after school enrollment, children with resolved pDSSD who have received speech therapy with PhonoSens exhibit comparable spelling skills to fourth-graders in the overall population in the German state of North Rhine-Westphalia in Germany.

**H1-3:**  The alphabetic spelling strategy particularly benefits from PhonoSens treatment and leads to better test scores than the other spelling strategies (orthographic, morphemic, and across-word).

**H1-4:**  The risk factors of parental education, familial risk for DLD, gender, and baseline phonological working memory skills have predictive value for later spelling competence.

**H2:**  Children with resolved pDSSD who have received speech therapy with PhonoSens are not at an educational disadvantage and therefore receive a comparable secondary school recommendation as children from the general population of the overall county population.

## 2. Materials and Methods

### 2.1. Participants

The study presented here is a follow-up study to a randomized controlled trial (RCT) on the effectiveness of the PhonoSens integrated treatment for pDSSD, which was conducted in German-speaking children with isolated pDSSD [49]. Thirty-two children (14 boys, 18 girls, median age 4.6 years, range 3.6 to 5.5 years) participated in the RCT. Age-appropriate development and normal hearing (ensured by pediatricians’ preventive examinations or otolaryngologists), and an isolated pDSSD with no other language deficits, confirmed by speech-language tests by speech therapists, were considered inclusion criteria in the RCT. Structural language problems due to speech-motor, anatomic, or intraoral sensory difficulties and neurologic disorders were exclusion criteria. The children were randomly assigned to a treatment group (median age 4.6 years, range 3.6 to 5.5 years) or a wait-list control group (median age 4.6 years, range 3.8 to 5.1 years). The gender imbalance was equal across groups (nine female, seven male). Initial target testing at study inclusion (naming-test) was followed by a period during which the 16 children in the treatment group received 15 weekly PhonoSens treatment sessions of 45 min each. After 15 sessions, their speech sound production was reassessed. Children who still had pDSSD continued to receive treatment. The speech sound production of the 16 children in the wait-list control group was also tested at the end of an equivalent waiting period. As a result, one child no longer needed treatment (according to the therapist’s assessment), and the parents of two children no longer thought the treatment was necessary. One child discontinued treatment after seven sessions. The 12 remaining children from the wait-list group completed their 15 weekly treatment sessions and were retested. After 15 treatment sessions for the treatment group and an equivalent waiting period without treatment for the wait-list control group, the treatment group showed significantly greater progress in percent correct consonants (PCC) and significantly greater decline in phonological processes than the wait-list control group, both with large effect sizes (Cohen’s d = 0.89 and 1.04). The progress of children in the wait-list control group during their therapy phase, which followed the waiting period, was comparable to the progress formerly measured in the treatment group after their therapy phase. All 28 children who underwent treatment overcame their pDSSD after 15–66 sessions (mean = 28, SD = 14.6), 21 of them before regular enrollment in elementary school and the other seven during first grade. 

The follow-up study described here began three to six years after successful completion of treatment and focuses specifically on the spelling acquisition of the participating children. The parents of the formerly treated 28 children were invited to participate with their child in a follow-up assessment to evaluate their children’s spelling skills at least three years after completion of treatment. Twenty-six parents (93.9%) gave written consent for their children to be tested, and their children gave verbal approval. One child refused to participate and one family was unable to attend for personal reasons. Fifteen girls and eleven boys participated, aged 9.3 to 11.2 years (mean = 10.1), at the end of the third to sixth grade. All participants attended regular schools. No participant was identified as having special educational needs. The educational level of the parents was distributed as follows: Twelve of the twenty-six participating children had at least one parent with a higher level of education (≥12 years), and fourteen children had at least one parent with a middle school education (10 years of education). More details are found in Table 1 and in Appendix A (Table A1).

The study has been approved by the Ethical Board of the Medical Faculty at the Goethe University Frankfurt am Main, Germany, registration number 313/10.3. 

### 2.2. Method

The spelling competencies for individual words and sentences of the 26 participating children were assessed at the end of their grade with the Hamburg Spelling Test (HSP) [25].

The HSP is a psychometric test with school-form and class-specific norms that is consistent with the recommendations of a German interdisciplinary and evidence-based guideline on literacy deficits [51]. Testing was conducted by two independent speech-language therapists who were not familiar with the children, their treatment outcome, or their outcomes in the previous RCT. The test analysis was carried out using the online tool from the test publisher. Every test has been administered by only one examiner. Since it was based on scoring and not on a rating, no interrater reliability was assessed. The test-retest reliability and inter-examiner reliability for the HSP has been reported by its authors in the test manual [25]. The test submission in the online tool was reviewed by a second independent speech-language pathologist. No inconsistencies were found.

Using the online tool, the number of correctly written words and the number of correct graphemes are recorded, and an analysis of spelling strategies is conducted. The criterion for impaired spelling ability is a discrepancy of 1.0 SD or more (T values < 40) below the mean of the class norm for the main test values: number of correctly written words or number of correct graphemes. Capabilities and strategies assessed in the HSP include the ability to translate spoken phonemes into written graphemes (alphabetic strategy), orthographic awareness (orthographic strategy), morphological awareness (morphemic strategy), and, from fourth grade onwards, the across-word spelling strategy (which derives orthographic rules from the context of the word and sentence).

The recommendation by the elementary school for each child to attend a secondary school was additionally surveyed as an indicator of long-term educational success. For children tested at the end of third grade, the secondary school recommendation was assessed one year later, at the end of fourth grade.

### 2.3. Outcome Measures

HSP scores are reported as T-values (10 points = 1 standard deviation). T-values less than 40 (>1 SD below the mean) represent deficient performance in the HSP. For all children, the number of correctly written words and the number of correct graphemes, and for the spelling strategies, the number of correctly used alphabetic, orthographic, and morphemic markers is assessed. In addition, the number of correctly used across-word markers is determined for children from fourth grade and above [25].

The individual number of correctly-written words, of correct graphemes, and the scores for the spelling strategies in the HSP (alphabetical, orthographic, morphemic, across-word) were recorded as a measure of spelling success in our study (for individual scores see Appendix B, Table A2).

Pretreatment baseline data from the earlier PhonoSens RCT were used to identify early predictive associations with later spelling success: (1) parental education level, (2) family predisposition to DLD, (3) gender, and (4) baseline scores of the former RCT on the subtest ‘phonological working memory for non-words’ of the German Speech Development Test for 3–5-year-old Children (SETK 3–5) [47] (individual scores are found in Appendix B, Table A2). For parental educational level, each parent was assigned 0–3 points for his or her education level: 0 = less than 10 years of education or special education, 1 = 10 years of low level education, 2 = 10 years of middle education, 3 = at least 12 years of higher education. The values of both parents were summed.

In Germany, different levels of qualification are available at the end of each of the different types of secondary school. Therefore, at the end of the fourth grade, every child receives a recommendation for subsequent attendance at one of the two secondary school forms (a) middle school (10 school years) or (b) high school (12–13 school years). This elementary school recommendation is therefore an indirect predictor of likely future educational attainment.

### 2.4. Statistical Analysis

The number of children with spelling deficits was descriptively compared to the number of children found to have a spelling disability in the study by Schnitzler et al. [27], which examined the spelling abilities of children whose pDSSD was resolved by unspecified speech therapy by the time of school enrollment. The number of children with spelling deficits identified here also was compared descriptively with the expected number based on the results of a German education report, according to which 22.1% of 29,259 fourth-graders did not meet the minimum standards (>1 SD below the mean) in spelling performance [12].

Further descriptive analysis determined the number of children with deficient profiles in the application of the different spelling strategies (alphabetic, orthographic, morphemic, across-word).

A multiple linear regression analysis was used to examine how well the predictors, parental education level, family predisposition to DLD, gender, and pretreatment T-values for phonological working memory for non-words anticipated the spelling proficiency, and the contribution of each predictor.

## 3. Results

### 3.1. Spelling Skills

Three of the twenty-six participating children (Table 2) performed below average (T-scores in the HSP below 40 for the number of correctly written words or for the number of correct graphemes) and had thus, by definition, a spelling disorder. No child performed poorly on the alphabetic spelling strategy. Five children each scored below average on the orthographic and morphemic spelling strategies. Children in fourth grade and above were also tested for the across-word spelling strategy: only one child out of seventeen demonstrated deficient performance.

The distribution of participants across their grade levels and the number of participants with deficient spelling performance per grade are displayed in Table 3. The spelling performance was determined to be insufficient in 3 out of 26 children (11.5%).

Schnitzler’s study [27] found spelling deficits in 56% of children (n = 32) with resolved isolated pDSSD (grades 1–4), a far higher proportion, which would be equivalent to 14.6 children in our study (grades 3–6). The percentage of children with spelling deficits in our sample is even lower than the 22.1 % of the population-based sample of fourth-graders (reflecting the typical proportion of children with resolved pDSSD) reported by the German education study for the general population in Germany [12] (equivalent to 5.7 children in our study).

### 3.2. Multiple Regression

We fitted a multiple linear regression model with the T-value for the number of correctly written words in the HSP as the continuous outcome variable and the covariates parental education level, family risk, gender, and phonological working memory for non-words (PWM), with the first three considered as factors with six, two, and two levels, respectively. Table 4 displays the ANOVA table for the fitted model.

The prediction of spelling ability revealed significant effects of parental education level (F(5) = 2.9, *p* = 0.044) and family risk (F(1) = 5.9, *p* = 0.027), while gender (F(1) = 0.0041, *p* = 0.95) and phonological working memory for non-words (PWM) did not yield a significant effect (F(1) = 1.8, *p* = 0.20).

### 3.3. Educational Success

Table 5 shows a comparison between the elementary school recommendations regarding secondary schooling after fourth grade received by study participants and the actual distribution of fifth-grade attendance at secondary schools in the school district of the study region for the same period. Eleven out of the twenty-six participants in our study (42.3%; five girls, six boys) received a recommendation to attend high school (12–13 school years duration), and 15 children (57.7%; 10 girls, 5 boys) middle school (10 school years duration). In comparison, 34.7% of fourth-graders in the local school district went to a secondary school, 64.6% to a middle school, and 0.6% to other schools in the same period [52].

## 4. Discussion

A (resolved) pDSSD is generally considered a particular risk factor for spelling development since it is assumed that underlying cognitive-linguistic deficits resulting from inadequate phonological language processing [5,50] may impede the development of written language acquisition [6]. Unfortunately, follow-up studies in general, and especially for German-speaking children with resolved pDSSD, are rare and often lack information regarding the previously-used therapy methods. In the study presented here, all 26 children were treated with the same therapy method—PhonoSens—and their spelling skills were assessed at least three years after the successful completion of therapy with PhonoSens [49]. All children visited regular classes. Only 11.5% of them showed a spelling deficit, a proportion that is considerably less than the 56% found in a similar population by Schnitzler [26,27], and even less than the 22.1% of fourth-graders with spelling deficits in the general population [12]. These results suggest that a phoneme-sensitive treatment approach, with its focus on improving internal and external auditory self-monitoring skills, may be associated with positive spelling development in the long term. This is all the more remarkable given that the long-term therapeutic effects of DLD remain unsatisfactory, as evidenced by a recent meta-analysis [53].

Methods that promote sensitivity to single phonemes are expected to have a positive impact on spelling development [26,38]. However, there may be other aspects of treatment for pDSSD that have a positive impact on later spelling development. The PhonoSens approach focuses on the integration of phonological and phonetic processing according to the Integrated Psycholinguistic Model of Speech Processing (IPMSP) [54]. The phoneme sensitivity of the PhonoSens method is achieved by teaching the child clearly distinguishable phoneme contrasts for the target phoneme and the error phonemes used, reinforcing the identification of individual phonemes, and linking phonemes to speech sound symbol pictures (SSP cards). In addition, from the beginning of therapy, PhonoSens focuses on improving internal self-monitoring skills (detection of incorrectly-planned phonemes) and external auditory self-monitoring skills (error correction and instant articulatory adaptation) for self-produced speech (for details of this method see Siemons-Lühring et al. 2021, [49]). 

One indication that treating pDSSD in this way can have a positive impact on spelling development is the evaluation of the alphabetic spelling strategy (write as you speak). For all participants in the present study, the T-values for the alphabetic spelling strategy showed standard or above-average values ranging from 41 to 64. This positive result might also be facilitated by the alignment of spelling education in the first years of elementary school. In the German state of North Rhine-Westphalia, where the present study was conducted, spelling education starts with “write as you speak”. Children with resolved pDSSD may have benefited from this approach. However, the influence seems to be limited, as the School Education Report for North Rhine-Westphalia shows a rate of 23.9% for spelling deficits in fourth-graders, which is even higher than for the overall population of fourth-graders in Germany [12].

Another indication that the applied therapy method might have had a positive effect on the alphabetic strategy is the comparison with the test scores for the other spelling strategies. Only in the alphabetic strategy, no child showed deficient performance. For the orthographic and morphemic strategies, 5 of 26 (19.2%) children each performed below average, and for the across-word strategy, 1 of 17 children (5.9%) demonstrated inadequate performance. We therefore tentatively suggest that the treatment approach used, rather than the learning method, may have had a positive effect on the development of the alphabetic spelling strategy.

Another factor influencing later spelling abilities could be the nature of the prior (isolated) pDSSD, i.e., as either a phonological delay characterized by persistent physiological phonological processes with consistent word production (which, however, is a special type of phonological disorder) or phonological disorder with pathological phonological processes and consistent or inconsistent word production (with both of these characterizations stemming from the application of one common classification [5]). In the study by Schnitzler [27], the children with resolved isolated pDSSD were further divided into children with resolved phonological delay (n = 10) or with resolved phonological disorder (n = 22). A significant dependence on group membership was found (χ^2^ = 18.69, *p* < 0.001, Cramer’s V = 0.44) for the children with a spelling deficit: 30% of the children with resolved phonological delay (3 out of 10) and 68% of the children with resolved phonological disorder (15 out of 22) had impaired spelling competencies. Among the children presented here, only one child showed exclusively physiological processes (which is indicative of phonological delay). In all other children, at least one phonological process error was classified as pathological [41]. Overall, three out of 26 children in this study showed deficits in spelling skills. Although both populations studied were relatively small—26 children with resolved isolated pDSSD in this study and 32 in Schnitzler’s study—the differences are substantial.

We hypothesized that the risk factors of parental education, the familial risk for DLD, gender, and basic phonological skills in working memory have predictive value for later spelling competence. However, the current multiple linear regression analysis suggests that only parental education and familial predisposition to DLD might influence spelling development and treatment outcome. Gender and phonological working memory for non-words (PWM) revealed no significant effect. In the previous RCT, no gender-specific effect was found for treatment progress after 15 treatments either. Boys appeared to benefit from therapy similarly to girls [49]. We hypothesize that this effect is reflected in their later spelling development, but the small group should be considered as a limiting factor.

Finally, one indicator of later educational success was examined. Long-term studies suggest that individuals with (resolved) pDSSD may be less successful in academic and occupational achievements and may have a lower social status later in life than individuals with typical phonological development [4,55]. In Germany, the first indicator of educational success is the elementary school recommendation regarding secondary school. There is a 10-year educational pathway leading to a middle school degree and a 12- or 13-year educational pathway leading to a high school qualification. The distribution of students in the local school district in the fifth grade was used as a population-based comparison group [52]. While approximately 35% of the fourth-graders in the overall county population transitioned to high school education, this was recommended for 42% of the participants in the study presented here. The educational success of the children studied here does not, therefore, appear to have been markedly affected by their resolved pDSSD.

## 5. Limitations

It was not possible to assemble a control group who had previously had pDSSD that had resolved without treatment. A comparison with children who underwent a different type of treatment for pDSSD than PhonoSens and whose baseline values and therapy results would have been evaluated retrospectively was not performed for technical reasons. Indirect group comparisons, therefore, had to be applied instead. 

The spelling tests used in the comparative studies were not applicable to our study due to the range of grade levels (third-sixth grade). In addition, further evaluation of the different spelling strategies was only possible with the HSP we used. The definition of poor performance was the same for all tests (1 SD below average). The final sample size of this longitudinal study was not particularly large.

Finally, the numerically lower rate of spelling deficits after PhonoSens treatment compared to prior studies could, in principle, also be affected by the period of formal spelling education. This is unlikely because the mean age of the participants of our study (10.1 years) approaches that one of the children assessed in the large population-based study we compared our outcome with [12].

## 6. Conclusions

Children with phonological developmental speech sound disorders have an increased risk of developing reading and spelling disorders at school age. Due to language specificities, these often manifest as spelling difficulties in German-speaking children. Our results suggest that, at least for German-speaking children, a phoneme-sensitive treatment method accompanied by an improvement in internal and external auditory self-monitoring skills, as applied in PhonoSens to preschool children with (isolated) pDSSD, may be associated with a positive long-term effect on these children’s later spelling competence and educational success. To our knowledge, this is the first longitudinal study that associates a positive relationship between the treatment outcome of a phonological intervention and spelling achievement in relation to school success. Parental education level and a family predisposition to DLD have a moderate influence on these outcome measures. Further studies with larger case numbers should replicate and substantiate these findings and extend them to other treatment modalities as appropriate.

## Figures and Tables

**Table 1:** Distribution of parents’ educational level across children for the present study (n = 28) and for the former RCT (n = 32) [49].

Educational Level of Both Parents	Σ Educational Level *	#Children [%] PRES. Study (n = 26)	#Children [%] former RCT (n = 32)
Both with high level education	6	3 [11.5]	3 [9.4]
One with high, one with middle level education	5	8 [30.8]	9 [28.1]
One with high, one with low level education	4	1 [3.8]	1 [3.1]
Both with middle level education	4	4 [15.4]	7 [21.9]
One with middle, one with low level education	3	5 [19.2]	6 [18.8]
Both with low level education	2	3 [11.5]	4 [12.5]
One with low level, one with special/<10 years of education	1	2 [7.7]	2 [6.3]
Mean education level		3.88	3.84

* Each parent was assigned 0–3 points for his or her education level: 0 = less than 10 years of education or special education, 1 = 10 years of low level education, 2 = 10 years of middle education, 3 = at least 12 years of higher education (values of both parents summed).

**Table 2:** Number of participants in the total number of children tested with T-scores below 40 for the main test values of the HSP and for the spelling strategies: correctly written words (#Corr. Writ. W.), number of correct graphemes (#Corr. Graph.), and the spelling strategy scores (alphabetical = Alph. Strat., orthographic = Orth. Strat., morphemic = Morph. Strat, and across-word = Acr.-W. Strat.).

#Corr. Writ. W.	#Corr. Graph.	Alph. Strat.	Orth. Strat.	Morph. Strat.	Acr.-W *. Strat.
3/26	3/26	0/26	5/26	5/26	1/17

* Only children from fourth grade and above.

**Table 3:** Distribution of participants (children with resolved isolated pDSSD) across their grades as well as the number (#) of participants with deficient spelling performance per grade.

Grade	# Participants per Grade	# Participants with Spelling Deficits
3rd	9	1
4th	12	2
5th	4	0
6th	1	0
3rd–6th	26	3

**Table 4:** ANOVA table for the fitted multiple regression model.

	Df	F Value	*p* (>F)
Factor (edu state parents)	5	2.9194	0.04417
Factor (family risk DLD)	1	5.8957	0.02657
Factor (gender)	1	0.0041	0.94963
T-Value PWM	1	1.8134	0.19579
Residuals	17		

**Table 5:** Elementary school recommendations received by participants in the present study (PhonoSens RCT follow-up) for secondary schooling after fourth grade compared with the actual distribution of fifth-grade attendance at secondary schools in the school district of the study region for the same period.

	Participants in the Present Study (n = 26)	Fifth-Graders within the Regional School District (n = 4018)
middle school	57.7%	64%
high school	42.3%	34.7%
other	0%	0.6%

## Data Availability

All data used to support the finding of this study are available from the corresponding author upon request. The data are not publicly available because they concern the privacy of children.

## Appendix A

**Table A1:** Individual baseline values from the former RCT for parental education level *, and familial predisposition to developmental language disorders (DLD; 1 = Y, 2 = N), Gender (1 = male, 2 = female), and T-values for pretreatment phonological working memory for non-word repetition (PWM).

Participant	Parental Education Level	Family Risk of DLD	Gender	T-Value PWM Pretreatment
PH-001	4	1	2	41
PH-002	5	1	2	29
PH-003	4	1	2	29
PH-004	3	2	2	35
PH-005	4	1	2	35
PH-007	5	1	2	47
PH-008	6	1	1	32
PH-010	5	2	2	40
PH-011	6	2	2	39
PH-012	3	1	2	35
PH-013	3	1	1	37
PH-014	4	2	1	39
PH-016	2	1	2	23
PH-017	6	1	2	40
PH-018	5	1	2	29
PH-019	5	2	1	37
PH-020	3	2	2	37
PH-022	5	2	1	37
PH-023	1	1	1	23
PH-024	3	2	1	29
PH-025	1	2	1	37
PH-026	2	1	2	31
PH-027	2	1	2	35
PH-028	5	2	1	45
PH-031	4	1	1	41
PH-032	5	1	1	27

* Each parent was assigned 0–3 points for his or her education level: 0 = less than 10 years of education or special education, 1 = 10 years of low education, 2 = 10 years of middle education, 3 = at least 12 years of higher education. The values of both parents were summed.

## Appendix B

**Table A2:** Individual T-values for the subtests of the HSP: correctly written words (#Corr. Writ. W.), number of correct graphemes (#Corr. Graph.), and the spelling strategy scores (alphabetical = Alph. Strat., orthographic = Orth. Strat., morphemic = Morph. Strat, and across-word = Acr.-W. Strat.).

			T-Values			
Participant	#Corr. writt. w.	#Corr. graph.	Alph. strat.	Orth. strat.	Morph. strat.	Acr.-W.* strat.
PH-001	41	41	47	35	50	
PH-002	56	58	59	52	58	56
PH-003	40	40	61	41	38	46
PH-004	48	52	51	48	46	56
PH-005	35	36	46	34	38	41
PH-007	56	57	57	60	63	
PH-008	44	45	46	40	53	46
PH-010	48	48	47	45	55	
PH-011	58	55	46	56	53	56
PH-012	32	34	47	32	34	
PH-013	43	43	44	47	40	61
PH-014	52	52	57	60	50	
PH-016	44	47	64	50	39	61
PH-017	58	64	59	63	46	56
PH-018	51	52	47	45	50	
PH-019	54	58	51	52	58	56
PH-020	54	55	51	56	49	56
PH-022	48	52	42	52	58	46
PH-023	39	39	42	39	43	30
PH-024	52	52	57	52	63	
PH-025	40	41	51	39	34	41
PH-026	45	44	53	45	41	47
PH-027	54	55	44	73	51	40
PH-028	66	67	57	60	63	
PH-031	47	49	51	48	49	56
PH-032	45	46	41	60	44	

* Only children from fourth grade and above.
